# Association of Histones With Coagulofibrinolytic Responses and Organ Dysfunction in Adult Post-cardiac Arrest Syndrome

**DOI:** 10.3389/fcvm.2022.885406

**Published:** 2022-06-28

**Authors:** Asumi Mizugaki, Takeshi Wada, Takumi Tsuchida, Satoshi Gando

**Affiliations:** ^1^Division of Acute and Critical Care Medicine, Department of Anesthesiology and Critical Care Medicine, Hokkaido University Faculty of Medicine, Sapporo, Japan; ^2^Department of Acute and Critical Care Center, Sapporo Higashi Tokushukai Hospital, Sapporo, Japan

**Keywords:** histone, disseminated intravascular coagulation, post-cardiac arrest syndrome, coagulation, fibrinolysis, multiple organ dysfunction syndrome

## Abstract

**Background:**

Patients successfully resuscitated from cardiac arrest often develop organ dysfunction caused by systemic inflammation and increased coagulation, leading to disseminated intravascular coagulation (DIC). The involvement of histones in DIC and organ dysfunction in patients with sepsis and trauma has been previously reported, raising the probability that histones may also be associated with pathophysiology in patients after cardiac arrest and resuscitation. This study evaluated the relationship between histones and organ dysfunction related to coagulofibrinolytic changes in patients with post-cardiac arrest syndrome (PCAS).

**Methods:**

This prospective single-center observational study assessed 35 adult patients with PCAS who were divided into two groups, i.e., 15 patients with multiple organ dysfunction syndrome (MODS) and 20 patients without MODS. MODS was defined as a sequential organ failure assessment score of ≥12. The plasma levels of histones and coagulofibrinolytic markers, including soluble fibrin, tissue-type plasminogen activator, plasminogen activator inhibitor-1, plasmin-alpha 2-plasmin inhibitor complex (PIC), and soluble thrombomodulin, were measured in patients with PCAS immediately after admission to the emergency department, and 3 and 24 h after arriving at the hospital.

**Results:**

PCAS patients with MODS had higher DIC scores [4 (3.0–5.0) vs. 1 (0.0–3.0), *p* = 0.012] and higher mortality rates (66.7% vs. 20.0%, *p* = 0.013) than those without MODS. Moreover, patients with MODS exhibited higher histone levels than those without MODS during the early phase of the post-resuscitation period. Severe endothelial injury and higher thrombin and plasmin generation were observed in the MODS group. Plasma levels of histones were positively correlated with those of soluble fibrin immediately after resuscitation (rho = 0.367, *p* = 0.030) and PIC 3 h after arriving at the hospital (rho = 0.480, *p* = 0.005). This correlation was prominent in the patient population with MODS (soluble fibrin: rho = 0.681, *p* = 0.005, PIC: rho = 0.742, *p* = 0.002).

**Conclusions:**

This study demonstrated that elevated histone levels were associated with increased levels of thrombin, and subsequent plasmin generation in PCAS patients, especially those with MODS. Further studies are required to elucidate the causal relationship between histones and organ dysfunction related to DIC in PCAS.

## Introduction

Over the past half-century, the rate of return of spontaneous circulation (ROSC) has considerably improved owing to the widespread use of cardiopulmonary resuscitation (CPR) among the public and advances in treatment methods for patients after cardiac arrest. However, the long-term survival of resuscitated patients remains poor. The high mortality rate in patients who are successfully resuscitated after cardiac arrest may be ascribed to the development of post-cardiac arrest syndrome (PCAS) (1). The International Liaison Committee on Resuscitation published a consensus statement on PCAS, characterized by four key components, i.e., (i) post-cardiac arrest brain injury, (ii) post-cardiac arrest myocardial dysfunction, (iii) systemic ischemia/reperfusion response, and (iv) persistent precipitating pathology (1). The pathophysiology of systemic ischemia/reperfusion response includes systemic inflammatory response syndrome and increased coagulation leading to disseminated intravascular coagulation (DIC) (2, 3). Various studies have proposed the following essential disease conditions for DIC: generalized inflammatory responses with the release of inflammatory cytokines and intravascular activation of coagulation with loss of localization that can both originate from and cause damage to the microvascular endothelium (4, 5). Several studies support the notion that sepsis, trauma, or cancer, which have long been recognized as major underlying causes of DIC, and PCAS could give rise to DIC (2, 3, 6, 7). Endothelial cell damage by ischemia/reperfusion in patients with PCAS can lead to coagulation abnormalities, consequently leading to further damage to endothelial cells and the subsequent consumption and exhaustion of platelets and coagulation factors (4). Furthermore, impairment of natural anticoagulant pathways and inhibition of fibrinolysis can exacerbate DIC in patients with PCAS (3). If these changes are sufficiently severe, DIC can induce multiple organ dysfunction syndrome (MODS) through systemic microvascular thromboses, affecting patient outcomes (4, 5, 8, 9). In particular, microcirculatory disorder in the brain caused by DIC, referred to as the “no-reflow phenomenon,” may be associated with post-cardiac arrest brain injury, which is the leading cause of mortality in patients with PCAS (3, 10). These findings remind us that control of DIC associated with PCAS may improve patient outcomes.

Recent studies have shown the importance of bidirectional interactions between inflammation and coagulation that are triggered by damage-associated molecular patterns (DAMPs), such as histones— in response to cellular injury and neutrophil extracellular traps (NETs) released by activated neutrophils—in the pathophysiology of DIC (3, 8). Although some studies have examined the relationship between histones and coagulopathy in sepsis (11, 12) and trauma (13), the association between histones and PCAS pathophysiology has not yet been examined. Therefore, we evaluated the association of histones in the pathomechanisms of organ dysfunction related to the coagulofibrinolytic responses associated with PCAS.

## Materials and Methods

### Study Design and Patients

We conducted a single-center prospective observational study. A total of 47 patients who experienced out-of-hospital cardiac arrest were enrolled between September 2018 and June 2020. Patients were excluded if they did not achieve ROSC or were under 20 years of age. Written informed consent was obtained from the patients' next of kin to draw blood samples and record essential data necessary for scoring DIC, acute physiology and acute chronic health evaluation (APACHE) II, and sequential organ failure assessment (SOFA) scores. If the next of kin did not accompany any of the patients, informed consent was retrospectively obtained after collecting blood samples at 0 h. In addition, blood samples from 15 healthy volunteers [median age of 29 (range 20–45), male: 53.3%], who were medical students and medical staff in our hospital), were collected to measure plasma levels of histones and coagulofibrinolytic markers. The study was approved by the Institutional Review Board of the Ethics Committee of Hokkaido University School of Medicine (017-0518).

### Definition

DIC was diagnosed according to the Japanese Association for Acute Medicine (JAAM) DIC score (Supplementary Table 1) (14). The APACHE II score was used to assess disease severity (15). The SOFA score was used to evaluate organ dysfunction (16). MODS was defined as a SOFA score of ≥12 (17). SOFA scores were measured on days 0, 1, and 3 after ROSC. Patients who achieved a SOFA score of ≥12 at least once were assigned to the MODS group.

### Data Collection

Blood samples were collected from an arterial catheter in patients using a syringe at three-time points: immediately after ROSC (0 h), 3 h after ROSC (3 h), and 24 h after ROSC (24 h). Blood samples from healthy volunteers were collected once per person. Blood samples were immediately placed into tubes with sodium citrate and centrifuged at 1,500 × g for 15 min at 4°C. The plasma was stored at −80°C until further examination.

We measured the plasma levels of histones (Histone H3 ELISA; Shino-Test Corp., Tokyo, JAPAN) (18) and the following coagulofibrinolytic molecular markers: (i) soluble fibrin (SF) as a marker of thrombin action on fibrinogen (LA, IATRO SFII; LSI Medience Corp., Tokyo, JAPAN); (ii) tissue-type plasminogen activator (t-PA) as a marker of t-PA release from endothelium (Human tPA ELISA kit; Innovative Research, Inc., Novi, MI, USA); (iii) plasminogen activator inhibitor-1 (PAI-1) as a marker of inhibition of fibrinolysis (LA, LPIA-tPAI test; LSI Medience Corp.); (iv) plasmin-alpha 2-plasmin inhibitor complex (PIC) as a marker of plasmin generation (LPIA, LPIA-ACE PPI II; LSI Medience Corp.); and (v) soluble thrombomodulin (sTM) as a marker of endothelial injury, with its measurement likely including extracellular vesicles shed from the endothelial plasma membrane (CLEIA, STACIA CLEEIA TM; LSI Medience Corp.). The tPA ELISA kit does not detect t-PA associated with PAI-1; it only detects free t-PA as supported by the protocol provided along with this kit specifying that functionally active tPA will form a covalent complex with biotinylated human PAI-1, which is bound to the avidin coated on the microtiter plate, and that complexed tPA will not bind to the PAI-1 and will not be detected by the assay. The reliability of these assay reagents has been verified, and the studies quantifying soluble fibrin measured using these reagents have been published (19–21).

Data necessary for scoring such as DIC score, SOFA score, APACHE II score, and other general coagulation markers, including antithrombin, fibrinogen/fibrin degradation products (FDPs), and D-dimer were retrieved from the medical records.

### Statistical Analysis

Numerical variables were presented as medians and quartiles, and categorical variables were summarized as numbers and percentages. Numerical data were compared among groups using the two-sided non-parametric Mann–Whitney *U*-test. Categorical data were compared among the groups using Fisher's exact test or Pearson's chi-squared test, depending on the size of the data. Correlations were evaluated using Spearman's rank test. Receiver operating characteristic (ROC) curve analyses with the area under the curve (AUC) calculation were used to quantify the predictive performance of the plasma levels of histones at each time point. Statistical significance was set at *P* < 0.05. All statistical analyses and calculations were performed using GraphPad Prism for Windows version 9.3.1 (Graph Pad Software, San Diego, CA, USA) and JMP Pro 16.0 (SAS Institute Inc., Cary, NC, USA).

## Results

### Characteristics of the Patients

Out of the 47 patients, 12 were excluded from the study. Among the excluded patients, 11 did not achieve ROSC. We also excluded one patient because the samples were not treated appropriately. In the present study, we analyzed the final cohort of 35 patients. The patients were divided into MODS (*n* = 15) and non-MODS (*n* = 20) groups. The characteristics of the patients in both groups are shown in Table 1. There were no significant differences in age, sex, or initial rhythm of the electrocardiogram between patients from the two groups. The frequencies of witnessing the onset of cardiac arrest and bystander CPR were comparable between groups. However, the non-MODS group had a significantly higher percentage of patients with cardiogenic cardiac arrests. The CPR duration was significantly longer in the MODS group than in the non-MODS group. Patients in the MODS group exhibited a higher DIC score on day 1 and a higher APACHE II score than those in the non-MODS group. Furthermore, the hospital survival rate was significantly higher in the non-MODS group than in the MODS group.

**Table 1:** Baseline characteristics of patients with PCAS with and without non-MODS.

	**Non-MODS** **(*n* = 20)**	**MODS** **(*n* = 15)**	***p*-value**
Age (year)	74.5 (66.0–80.3)	78.0 (63.0–82.0)	0.548
Gender: male (*n*, %)	12 (60.0%)	7 (46.7%)	0.433
Cause of cardiac arrest(*n*, %)			
Cardiogenic	15 (75.0%)	5 (33.3%)	0.019
Asphyxia	3 (15.0%)	5 (33.3%)	0.246
Central nervous system	1 (5.0%)	2 (13.3%)	0.565
Others	1 (5.0%)	3 (20.0%)	0.292
Shockable rhythm (*n*, %)	10 (50.0%)	4 (26.7%)	0.296
Witnessed arrest (*n*, %)	15 (75.0%)	9 (60.0%)	0.467
Bystander CPR (*n*, %)	10 (50.0%)	9 (60.0%)	0.557
CPR duration (min)	22 (16.0–27.0)	35 (28.0–37.0)	0.020
DIC score			
Day 0	2 (1.0–3.8)	3 (3.0–4.0)	0.821
Day 1	1 (0.0–3.0)	4 (3.0–5.0)	0.012
DIC (*n*, %)			
Day 0	5 (25.0%)	6 (40.0%)	0.467
Day 1	4 (21.1%)	9 (60.0%)	0.034
SOFA score			
Day 0	8 (6.5–9.3)	12 (9.5–12.5)	0.001
Day 1	7 (5.0–7.5)	11 (8.0–12.0)	<0.001
Day 3	6 (4.8–7.3)	12 (7.0–13.0)	0.006
APACHE II score	32.5 (27.0–37.0)	36.0 (33.0–41.0)	0.030
Outcomes survivor (*n*, %)	16 (80.0%)	5 (33.3%)	0.013

*APACHE II, Acute Physiology and Chronic Health Evaluation II; CPR, cardiopulmonary resuscitation; DIC, disseminated intravascular coagulation; MODS, multiple organ dysfunction syndrome; PCAS, post-cardiac arrest syndrome; SOFA, Sequential Organ Failure Assessment. Age, CPR time, DIC score, and APACHE II score were presented as medians and quartiles (lower upper). “Others” in “cause of cardiac arrest” were hanging in one case and unknown in three cases. DIC was diagnosed according to the Japanese association for acute medicine DIC diagnostic criteria*.

### Histones

**Figure 1:**
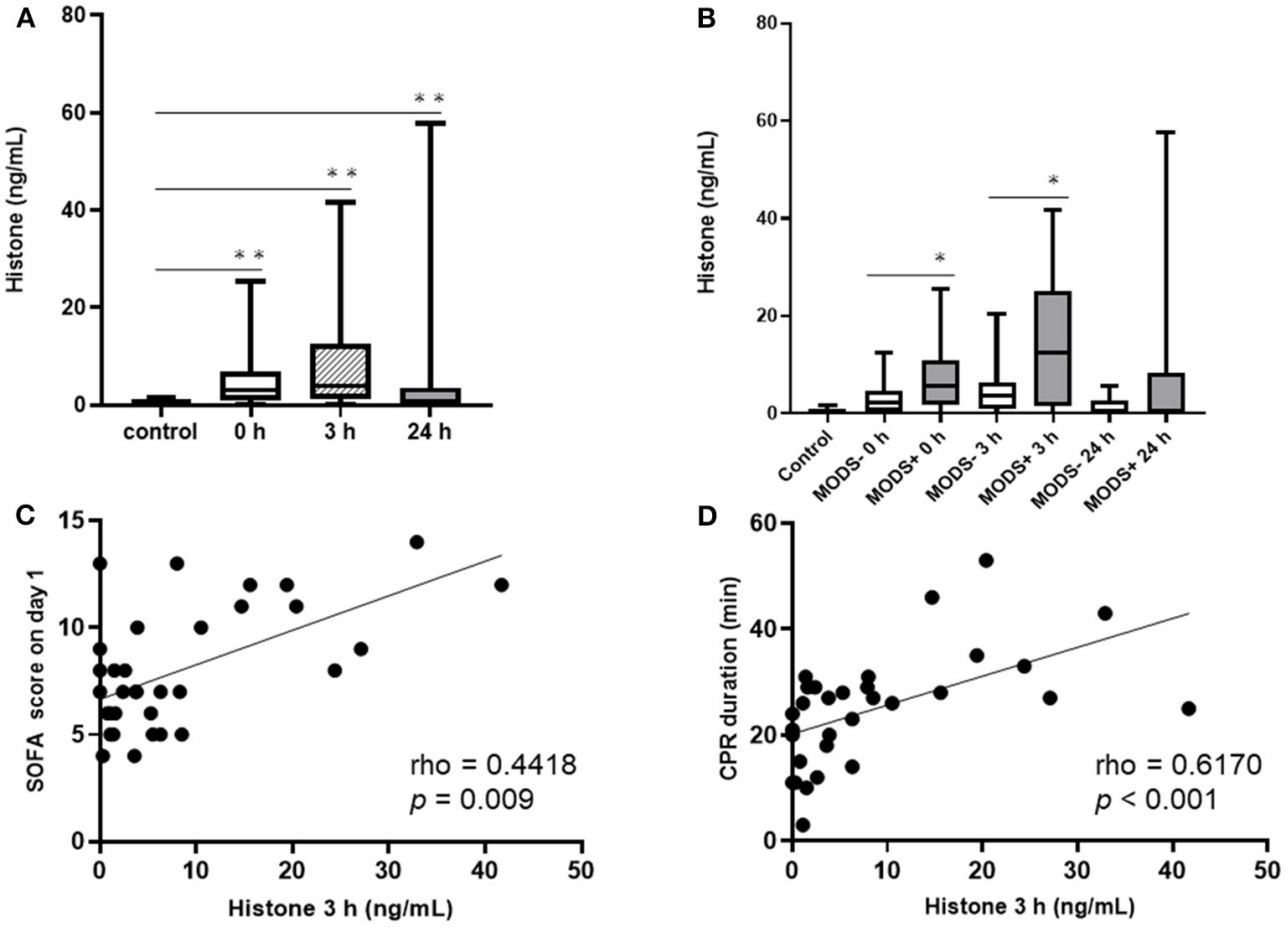
The serial changes of the plasma levels of histones and correlation between the levels of histones at 0 h and SOFA score on day 1 and CPR duration. **(A)** Serial change of the plasma levels of histones. **(B)** Changes in the plasma levels of histones in the MODS and non-MODS groups. **(C)** Correlation between the plasma levels of histones at 3 h and SOFA score on day 1. **(D)** Correlation between plasma levels of histones at 3 h and CPR duration. *p < 0.05, **p < 0.001. CPR, cardiopulmonary resuscitation; MODS, multiple organ dysfunction syndrome; SOFA, Sequential Organ Failure Assessment.

The plasma levels of histones in all participating patients were elevated at 0 and 3 h compared to those in the control subjects. Although the levels of histones were decreased by 24 h, they were still significantly higher than those in the control subjects (Figure 1A). We compared the plasma levels of histones between the groups with and without MODS (Figure 1B). Patients in the MODS group exhibited significantly higher levels of histones than those in the non-MODS group at 0 and 3 h. There was no significant difference in the plasma levels of histones between the two groups at 24 h. There was a positive correlation between the plasma levels of histones at 3 h and SOFA score on day 1 (Figure 1C). In addition, a positive correlation was found between plasma levels of histones at 3 h and CPR duration (Figure 1D).

### Coagulofibrinolytic Markers

Analysis of general coagulofibrinolytic markers for the two groups is shown in Supplementary Table 2. The median plasma antithrombin levels at 3 h were significantly lower in the MODS group than in the non-MODS group. However, the levels of fibrinogen/fibrin degradation products (FDPs) and D-dimer at 24 h were higher in the MODS group than in the non-MODS group.

**Figure 2:**
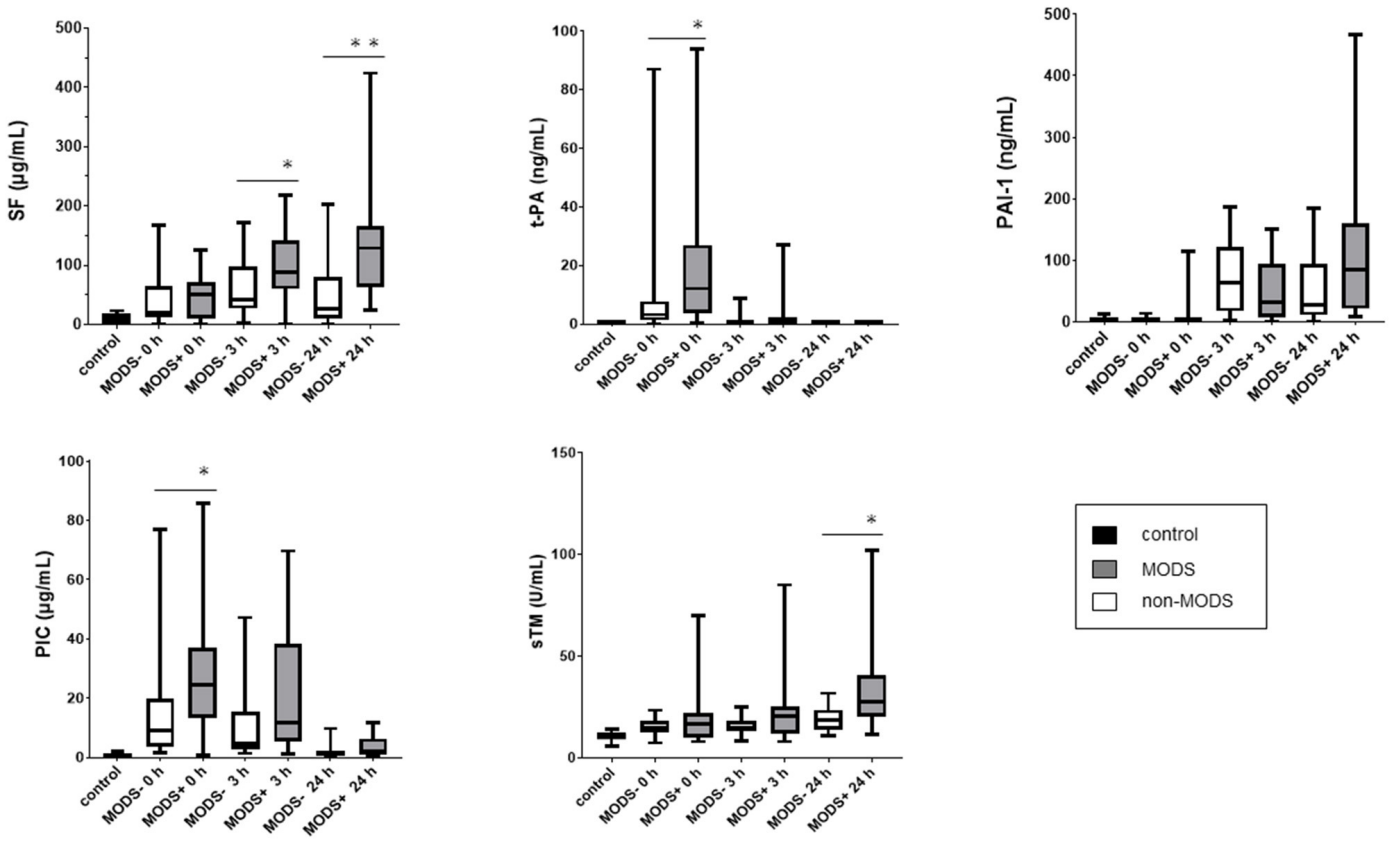
The serial changes of coagulofibrinolytic molecular makers in the MODS and non-MODS groups. *p < 0.05, **p < 0.01. MODS, multiple organ dysfunction syndrome; PAI-1, plasminogen activator inhibitor-1; PIC, plasmin-alpha 2-plasmin inhibitor complex; SF, soluble fibrin; sTM, soluble thrombomodulin; t-PA, tissue-type plasminogen activator.

Serial changes in coagulofibrinolytic molecular markers (SF, PIC, PAI-1, sTM, and t-PA) are shown in Figure 2. The SF and sTM levels in the MODS group were significantly higher at 24 h than those in the non-MODS group. In addition, plasma PIC and t-PA levels at 0 h were significantly higher in the MODS group than those in the non-MODS group, and there was a positive correlation between the plasma levels of PIC and t-PA at 0 h in the MODS group (rho = 0.930, *p* < 0.001). There were no significant differences in PAI-1 levels at any time point between the MODS and non-MODS groups, whereas PAI-1 levels at 24 h in the MODS group tended to be higher than those in the non-MODS group.

### The Relationships Between Histones and Coagulofibrinolytic Markers

We evaluated the correlation between plasma levels of histones and coagulofibrinolytic markers (Table 2). There was a positive correlation between histone and SF levels, measured at 0 h, when thrombin activation is considered to peak (3) in patients with PCAS (rho = 0.367, *p* = 0.030). This correlation was prominent in a population with MODS (rho = 0.681, *p* = 0.005). There was also a positive correlation between histone levels and PIC measured at 3 h, when the fibrinolysis is considered to peak (6, 7, 22), (overall: rho = 0.480, *p* = 0.005; MODS group: rho = 0.742, *p* = 0.002). This correlation became stronger in the MODS group.

**Table 2:** Correlation between histones and coagulofibrinolytic markers.

	**Overall**		**MODS**	
	**rho**	***p*-value**	**rho**	***p*-value**
Histone 0 h vs. SF 0 h	0.367	0.030	0.681	0.005
Histone 3 h vs. SF 3 h	0.280	0.115	0.141	0.6304
Histone 0 h vs. SF 0 h	0.105	0.549	0.118	0.6757
Histone 3 h vs. PIC 3 h	0.480	0.005	0.742	0.002

*MODS, multiple organ dysfunction syndrome; PIC, plasmin-alpha 2-plasmin inhibitor complex; SF, soluble fibrin*.

### Histones as a Predictive Marker for Developing MODS

**Figure 3:**
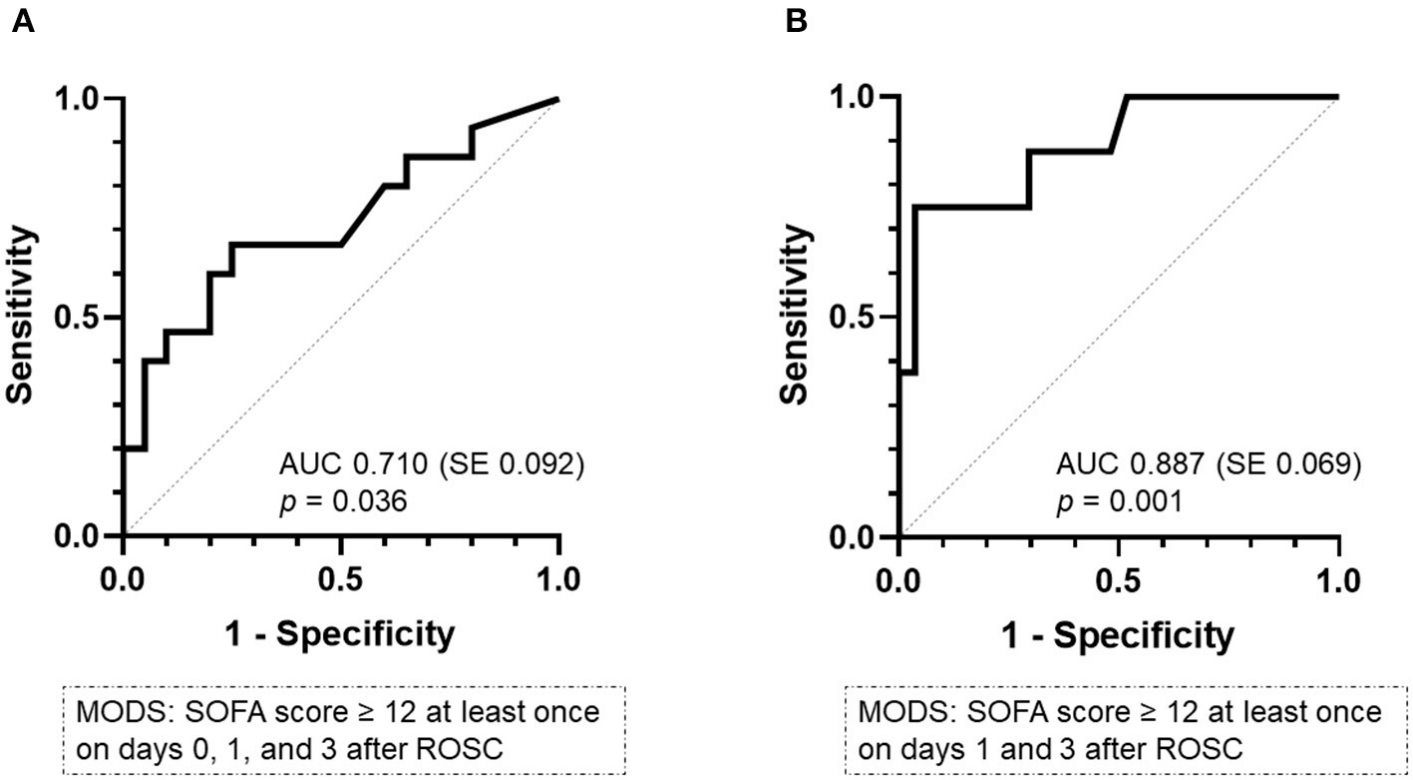
Receiver-operating characteristics curve analysis of the plasma level of histones at 0 h on predicting MODS. **(A)** MODS was defined as a SOFA score of ≥12 at least once on days 0, 1, and 3 after ROSC. **(B)** MODS was defined as a score of ≥12 at least once on days 1 and 3 after ROSC. AUC, area under the curve; MODS, multiple organ dysfunction syndrome; ROSC, return of spontaneous circulation; SE, standard error.

To quantify the predictive performance of plasma histone levels for the development of MODS, we analyzed receiver operating characteristic (ROC) curves. The area under the curve (AUC) for plasma levels of histones measured at 0 was 0.710 (standard error [SE] 0.092, 95% confidence interval [CI]: 0.529–0.891) (Figure 3A). In order to increase the clinical significance of the prediction of developing MODS using histones, the ROC analysis, in which patients with MODS were defined as those with a SOFA score of 12 or higher on days 1 and 3, was performed. The results showed higher performance of histones in predicting the development of MODS (AUC: 0.887, SE: 0.069, 95% CI: 0.752–1.000) (Figure 3B).

## Discussion

In the current study, we found that PCAS patients with MODS had higher DIC scores and higher mortality rates than those without MODS. In addition, patients with MODS exhibited massive thrombin and plasmin generation with higher levels of histones than those without MODS during the acute phase of PCAS. The levels of histones were positively correlated with SF, a thrombin generation marker, and PIC, a plasmin generation marker. According to our analysis, the plasma level of histones immediately after ROSC was a good predictor of MODS development in patients with PCAS.

Histones are commonly known as DNA-binding proteins that play an important role in compacting DNA to be stored in the nucleus (23–25). Histone toxicity is restricted when histones are present within the nuclei, but once released into the extracellular space, histones or DNA-histone complexes function as DAMPs. Histones are also a major component of NETs, acting as virulence factors against bacteria and other organisms; however, they can also cause cell damage (24).

A previous study demonstrated that the levels of circulating histones increase in mouse models of sepsis in a neutrophil-dependent manner, suggesting that histones are mainly derived from NETs (18). However, the present study demonstrated that there was a correlation between CPR duration and histone levels at 0 h (Figure 1D), indicating that the extracellular release of histones in patients with PCAS may be caused by the systemic tissue damage due to hypoxia and ischemia/reperfusion during cardiac arrest and resuscitation. This view is supported by a previous study that showed the involvement of histones and NETs in the pathomechanisms of PCAS-related DIC, including cytotoxicity, pro-coagulation, and pro-inflammation (7). During ischemia-reperfusion injury, cellular damage and the release of histones lead to a vicious cycle of thrombin generation owing to the interaction between histones and NETs. The present study indicated that hyperfibrinolysis as well as hypercoagulation occur in the early phase after cardiac arrest resuscitation, with histones being involved in this process. The positive correlation between histones and PIC may reflect hyperfibrinolysis caused by t-PA release from endothelial cells, and may involve the following mechanisms: (i) histone-induced thrombin stimulation, (ii) histone-induced endothelial cell damage, and (iii) profound hypoxic stimulation (5, 7, 26). These mechanisms may be supported by the differences in the effects of histones on coagulation and fibrinolysis depending on the time phase after ROSC (Table 2). Immediately after ROSC, blood t-PA levels increase, and a delayed decrease is observed owing to its binding to PAI-1, which is subsequently synthesized and secreted, referred to as “fibrinolytic shutdown” (2). The influence of PAI-1 after resuscitation can be inferred from the observation that the difference in t-PA values between the two groups became less significant over time. The cytotoxicity of histones causes vascular endothelial cell damage, which is reflected by an increased sTM levels (8, 13). This demonstrated that vascular endothelial cell damage persisted even after 24 h in the MODS group, resulting in organ damage. Although a causal relationship with the plasma levels of histones was not demonstrated in this study, it does show that procoagulation and hyperfibrinolysis followed by antifibrinolysis are apparent in conditions where histones are elevated.

Although this study cannot demonstrate a causal relationship between histones and the pathophysiology of PCAS-associated DIC, we believe that extracellular histones may be a promising therapeutic target for PCAS-associated coagulopathy and consequent organ dysfunction, as previous studies showed that the administration of neutralizing antibodies to histones can prevent death from severe sepsis in a murine model (27, 28). This study indicated the possibility that histones could be a promising marker to assess the severity of PCAS. In fact, extracellular histones have already been studied as potential diagnostic targets in sepsis and trauma (8, 11, 12, 29).

This study has several limitations. First, we excluded 11 patients without ROSC from initial cohort because this study aimed to elucidate the pathophysiology of PCAS. This may be a study limitation. Second, the sample size was small. This limitation may have resulted in wide variations in the measured parameters as shown in Figure 2. Furthermore, since the multivariate analysis was not performed owing to insufficient sample size, it was impossible to evaluate the complex relationships between coagulation and fibrinolysis markers. A multi-center population with large sample sizes should be evaluated in the future. Third, medical students and medical staff from our hospital were recruited as healthy volunteers using a selection method employed in our previous studies: however, they were not age- or sex-matched to the patients. Fourth, we centrifuged collected blood samples at 4°C to avoid false high value of measurements in accordance with our previous studies (19–21). However, one-time centrifugation of blood samples at low temperature without checking platelet counts can lead to residual platelets in the processed samples, which could have affected the measurements, especially PAI-1 values. Fifth, the reagent used to measure the soluble fibrin levels is available only in Japan. Sixth, we did not consider the influence of targeted temperature management, which is commonly performed on patients after achieving ROSC (1, 30, 31). In our institution, patients are usually kept hypothermic at 34°C or at normal body temperature at 36–37°C for 24 h after cardiac arrest resuscitation, but the effects of different body temperatures on patient outcomes could not be evaluated in this Study.

## Conclusion

To the best of our knowledge, this is the first study to investigate the relationship between histones and the coagulofibrinolytic changes associated with PCAS and to suggest the potential of histones for predicting the MODS development in patients with PCAS. Although the causal relationship between histones and organ dysfunction related to DIC in PCAS requires investigation, we expect that the neutralization of histones may represent a new therapeutic strategy for treating PCAS.

## Data Availability Statement

The raw data supporting the conclusions of this article will be made available by the authors, without undue reservation.

## Ethics Statement

The studies involving human participants were reviewed and approved by Institutional Review Board of the Ethics Committee of Hokkaido University School of Medicine (017-0518). The patients/participants provided their written informed consent to participate in this study.

## Author Contributions

AM analyzed the study results, interpreted the data, and drafted the manuscript. TW oversaw the entire research and contributed to the research concept, the analysis, and interpretation of the data, and was responsible for drafting, editing, and submitting the manuscript. TT contributed to the data acquisition, interpreted the data, and revised the manuscript for important intellectual content. SG had a significant influence on data interpretation and critical appraisal of the manuscript. All authors read and approved the final version of the manuscript before submission.

## Funding

This work was supported in part by JSPS KAKENHI [Grant-in-Aid (B) 2017, 17H04361].

## Conflict of Interest

The authors declare that the research was conducted in the absence of any commercial or financial relationships that could be construed as a potential conflict of interest.

## Publisher's Note

All claims expressed in this article are solely those of the authors and do not necessarily represent those of their affiliated organizations, or those of the publisher, the editors and the reviewers. Any product that may be evaluated in this article, or claim that may be made by its manufacturer, is not guaranteed or endorsed by the publisher.
