# Supplementary material for: Association of Histones With Coagulofibrinolytic Responses and Organ Dysfunction in Adult Post-cardiac Arrest Syndrome
Source: Front Cardiovasc Med. 2022 Jun 28;9:885406. doi: 10.3389/fcvm.2022.885406 (PMC9273886; doi:10.3389/fcvm.2022.885406)

Supplementary Material

**Supplementary Table 1**. Scoring system for disseminated intravascular coagulation (DIC) prescribed by the Japanese Association for Acute Medicine

----------------------------------------------------------------------------------------------------------------

1. Clinical conditions associated with DIC

1) Sepsis/severe infections (microorganisms)

2) Trauma/burn/surgery

3) Vascular abnormalities

Large Vascular Aneurysms

- giant hemangioma

- vasculitis

4) Severe toxicity or immunological reaction

- snakebite

- recreational drugs

- transfusion reactions

- transplant rejection

5) Malignancy (except for bone marrow suppression)

6) Obstetric calamities

7) Conditions that may be associated with SIRS.

- organ destruction (e.g., severe pancreatitis)

- severe hepatic failure

- ischemia/hypoxia/shock

- heat stroke/malignant syndrome

- fat embolism

- rhabdomyolysis

- other

8) Other

-----------------------------------------------------------------------------------------------------------

2. Clinical conditions that should be carefully ruled out.

A. Thrombocytopenia

1) dilution and abnormal distribution

Massive blood loss, transfusion, and massive infusion

2) increased platelet destruction

ITP, TTP/HUS, HIT, drugs, viral infection, alloimmune destruction, APS, HELLP, extracorporeal circulation.

3) decreased platelet production

Viral infection, drugs, radiation, nutritional deficiency (vitamin B12, folic acid), hematopoietic disorders, liver disease, HPS

4) spurious decrease

EDTA-dependent agglutinins and insufficient anticoagulation in blood samples

5) other

Hypothermia and artificial devices in vessels

B. Prolonged prothrombin time.

Anticoagulation therapy, anticoagulants in blood samples, vitamin K deficiency, liver cirrhosis, massive blood loss, and transfusion

C. Elevated FDP

Thrombosis, hemostasis and wound healing, hematoma, pleural effusion, ascites, anticoagulants in blood samples, antifibrinolytic therapy

D. Other

---------------------------------------------------------------------------------------------------------------

3. Diagnostic algorithm for SIRS

1) temperature > 38 °C or < 36 °C

2) heart rate > 90 bpm

3) respiratory rate > 20 breaths/min or PaCO_2_ < 32 torr (< 4.3 kPa)

4) White blood cell counts > 12,000 cells/mm^3^, < 4,000 cells/mm^3^, or 10% immature (band) forms.

---------------------------------------------------------------------------------------------------------------

4. The diagnostic algorithm

Score

SIRS criteria

≥3 1

0-2 0

Platelet count (10^9^/L)

<80 or greater than 50% decrease within 24 h 3.

≥80 or <120 or greater than 30% decrease within 24 h 1

≥120 0

Prothrombin time (value of patient/normal value)

≥1.2 1

<1.2 0

Fibrin/fibrinogen degradation product (mg/L)

≥25 3

≥10 <25 1

<10 0

Diagnosis

Four points or more DIC

-----------------------------------------------------------------------------------------------------------------

SIRS, systemic inflammatory response syndrome; ITP, idiopathic thrombocytopenic purpura; TTP, thrombotic thrombocytopenic purpura; HUS, hemolytic uremic syndrome; HIT, heparin-induced thrombocytopenia; APS, antiphospholipid syndrome; HELLP, hemolysis, elevated liver enzymes, and low platelets; HPS, hemophagocytic syndrome; EDTA, ethylenediaminetetraacetic acid; FDP, fibrin/fibrinogen degradation products

**Supplementary Table 2.** General coagulofibrinolytic parameters.

|  | Non-MODS (n=20) | MODS (n=15) | p-value |
| --- | --- | --- | --- |
| Platelets (10^9^/L) |  |  |  |
| 0 h | 189 (151–222) | 193 (156–233) | 0.701 |
| 24 h | 153 (122–205) | 180 (143–217) | 0.521 |
| PT-INR | | | |
| 0 h | 1.1 (1.1–1.3) | 1.2 (1.1–1.3) | 0.271 |
| 3 h | 1.2 (1.1–1.5) | 1.3 (1.1–1.6) | 0.825 |
| 24 h | 1.1 (1.1–1.2) | 1.2 (1.1–1.4) | 0.306 |
| Fibrinogen (g/L) | | | |
| 0 h | 2.4 (2.0–2.7) | 2.5 (2.3–3.1) | 0.414 |
| 3 h | 2.0 (1.8–2.5) | 2.4 (2.1–2.8) | 0.438 |
| 24 h | 2.5 (2.2–3.0) | 2.3 (2.1–2.8) | 0.555 |
| Antithrombin (AT, %) | | | |
| 0 h | 86.5 (73.8–94.3) | 77.0 (69.5–81.0) | 0.086 |
| 3 h | 83.0 (75.0–88.5) | 66.0 (57.0–76.0) | 0.022 |
| 24 h | 78.0 (68.5–94.0) | 73.0 (62.5–77.0) | 0.096 |
| FDP (mg/L) | | | |
| 0 h | 21.3 (9.9–48.5) | 41.9 (26.3–83.1) | 0.211 |
| 3 h | 31.0 (14.5–64.0) | 69.0 (26.2–233.4) | 0.152 |
| 24 h | 16.4 (6.7–30.8) | 51.5 (37.5–88.2) | 0.003 |
| D-dimer (mg/L) | | | |
| 0 h | 10.9 (6.1–23.3) | 18.4 (14.1–49.7) | 0.167 |
| 3 h | 14.8 (8.5–25.2) | 39.0 (17.7–128.3) | 0.084 |
| 24 h | 6.9 (2.8–11.5) | 20.8 (15.3–33.8) | <0.001 |

FDP, fibrinogen/fibrin degradation products; MODS, multiple organ dysfunction syndrome; PT-INR, prothrombin time-international normalized ratio.

Data are presented as median (interquartile range).


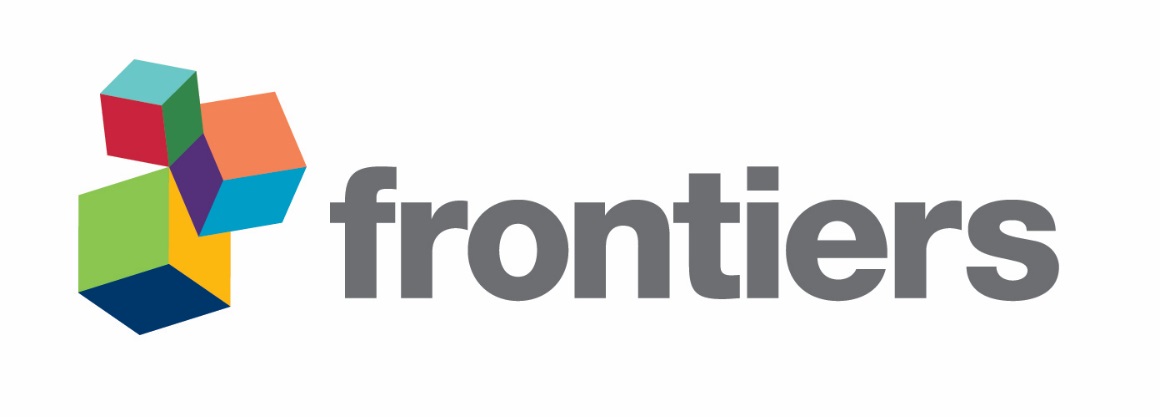

Supplement: Supplementary file 1 [file Data_Sheet_1.docx]
